# The Drug Drummer’s Story

**Published:** 1905-08

**Authors:** 


### TEXAS MEDICAL JOURNAL.

AUSTIN, TEXAS.

A MONTHLY JOURNAL OF MEDICINE AND SURGERY.

EDITED AND PUBLISHED BY
F. E. DANIEL, M. D.
Mrs. F. E. DANIEL, Business Manager.

**Published Monthly at Austin, Texas. Subscription price $1.00 a year in advance**

The contents of the "Red Back" are not copyrighted. They are intended for dissemination. Copy what you please, but give credit.

### THE DRUG DRUMMER'S STORY.

"Daniel," he said, as he wheeled the big Morris chair to the open south window, through which the fragrant breeze wafted a shower of apple blossoms, and the love notes and chirps of a pair of nesting sparrows were heard,—"notice how gray I have grown since I saw you last? Scared; I'll tell you about it." Helping himself to one of my 25-cent Havanas, that I keep for paying subscribers only, he told the following story:

### THE "HANT" WITH THE RED SCAR.

The doctor, a bachelor, aged fifty, sat in his cozy study, reading. It was Friday night. A fearful storm was coming up, and already the wind was howling and the shutters banging. It was as dark as Erebus. Deep and prolonged rumbling thunder was heard in the distance, and occasionally the sky was rent with a blinding lightning flash. I am a drug drummer, as you are aware. It is my business to visit all the doctors in every town in Texas and give them samples and literature, and talk up the products of the laboratory of Dope & Dosem. I am very fond of this particular doctor, and always visit him socially, after business hours, whenever I strike his town. The doctor laid down his book—it was Spencer's "First Principles"—motioned me to an easy chair, gave me a cigar and lighting one himself, squared himself for a talk.

I saw he was loaded, and I squared myself to receive the charge. He said:

"Spencer says that all beliefs, however absurd, have some foundation in fact; some germ of truth. I believe this is true. If many of the superstitious beliefs prevalent amongst the ignorant and credulous could be traced to their origin, I dare say that it would be found that some trifling incident perhaps, repeated—a mere coincidence—was magnified and handed down with exaggerations until a mole hill became a mountain. It has ever been the tendency of the human mind to attribute to supernatural causes phenomena that can not be explained. The ignorant believe in 'luck'—whatever that may be. We will suppose, to illustrate what I mean, that in days long gone by, several persons in a community had in succession, met with an accident on Friday, we will say. Soon Friday began to be looked upon as an 'unlucky' day—and it was 'unlucky' to do a thing or start on a journey on that day. We can readily understand that. It would be interesting to know what the germ of truth is, or was, that gave rise to the popular belief, especially prevalent in the South, that a cat has nine lives; or that the spirit of a wicked man, at death, enters the body of some beast, say a mule or a cat. Not only the ignorant, but many intelligent and cultivated persons subscribe to the doctrine of metempsychosis—the transmigration of the souls. I have seen a street car mule or a wagon mule overloaded and beaten, draw a deep sigh, as if to say: 'How long, oh Lord, how long?' and I can imagine that perhaps he is the reincarnation of a wife-beater, who now 'knows how it is himself,' and is undergoing the punishment that fits the crime. It is a more rational belief than a fire and brimstone hell, and there are those who say they believe that. In most instances such superstitious beliefs, I think, are the outgrowth of coincidences. That a cat has nine lives, and that the soul of a wicked man goes at death, into a cat, for instance, doubtless grew out of a series of coincidences similar, perhaps, to those I feel inclined to relate to you here and now. I have never told the story before because it is so weird and uncanny and improbable, that I fear I should be ridiculed; and, in fact, it gives me the creeps to recall it.

"This skeleton," said the doctor, rising and drawing back a heavy curtain, revealing a beautiful articulated skeleton in a glass case, "recalls the story every time I look at it. It is the skeleton of a young girl, with whom and a certain black cat, there is connected a remarkable story. It has, too, a peculiar fascination for a certain other large black cat, which made its appearance here simultaneously with the arrival of the skeleton from Philadelphia, where I had sent it to be articulated. I am much annoyed with this cat. I find him in here looking at the skeleton every time I return, and I have never been able to find out how he gets in. There he is now, behind you, peering at us through the window pane from the black darkness without. (I jumped and the doctor laughed at me.) Observe, he has but one eye. See how it gleams with green and gold from th black background."

It made cold chills run all over me. The cat's gaze fascinated me. I could not keep my eyes off of it. I listened with a creepy sensation to the doctor's story.

"I was taking a post-graduate course at the New Orleans Medical College last winter," said the doctor, "studying anatomy especially. The janitor informed me that there was a 'subject,' a 'lovely corpse,' he said, on my table. I was a most indefatigable dissector, and often remained in the dissecting room until 2 or 3 o'clock at night. It was Saturday night. The students do not dissect Saturday night. I was impatient to get at my fresh subject, and went alone to the gruesome work. Have you ever been in a dissecting room? No? Imagine twenty or thirty tables, each with a dead human body on it, in every stage of mutilation, and from hooks on the wall here and there, hang severed members—limbs, heads, ribs, all more or less dissected, a horrid fresco on which the pale electric light shines with a ghastly glare; while a score of cats of all degrees cry and growl in the empty corridors, or seated on the window sills at every wndow, glare at you with big, hungry, luminous eyes from the darkness without. Rather a cheerful environment for a man alone at midnight, eh? Ugh! It was a shock to my young nerves when I was a beginner."

As the storm grew nearer and louder the cat scratched frantically at the window pane, and gave a succession of loud, prolonged and distressed cries. It made my hair stand on end.

The doctor continuing, said:

"On entering the dissecting room I readily located my subject; the body of a young and remarkably well formed woman of about twenty. I observed, at a glance, that the only blemish on the marble surface of her beautiful body was a 'strawberry' birthmark on the left breast. Seated on her chest, staring into her face, was an enormous black boar cat! As he turned his gaze toward me, its baleful glare nearly unnerved me. He had but one eye, and it gleamed and blazed blue, green and flame colored, and shone like the headlight of a locomotive. I threw a thigh bone at him, and with a growl he escaped through a broken pane.

I had time to observe that besides being blind in his left eye, he had a hideous red scar across his face. Ugh! The devil couldn't have been uglier.

"I gave Pat, the janitor, $10 to prepare the skeleton for me when I had finished my studies on the body some weeks later.

" 'I sure earned that $10, Doctor,' said Pat to me when he delivered the bones, clean, dry and polished. 'There was a big, black devil of a cat,' he added, 'who determined to get at the body. I often throw scraps to the cats that assemble at the dead house while I am boiling a body to get the bones, and I am sorter used to them; but this fellow didn't eat the scraps. I couldn't drive him away. He'd fight me. At the little iron grated window overhead in the dead house he sat all the while I was working with the body, and boiling and cleaning the bones, and the gleam of his horrid right eye—his left was out—and that ghastly fiery red scar on his face, almost paralyzed me. I had to kill him, but I hated to,' said Pat, with a wag of his head.

"The bones were packed in straw in a cracker box and shoved under my bed. One night I had remained alone at the dissecting room later than usual, and was somewhat nervous, I suppose. My room was just in the rear of the college, on the second floor, front opening on the street, and the lamps below lighted it rather uncomfortably, notwithstanding the shutters and shades. I, for some foolish reason, or none, had put a large Bowie knife under my pillow. I couldn't tell you why—I am ashamed of it. I fell into a fitful doze, and was at once awakened by a rattling of the bones under the bed! I started up, each separate hair standing on end, and the cold sweat stood in beads on my face. I shivered. 'You are a fool,' I said to my self; 'bones can't move ,go to sleep; you are nervous and excited.' Laying my head again on the pillow, but (I couldn't help it), with my best ear cocked up in a listening attitude, soon again I was dropping off, when there came an unmistakable rattle of the bones, louder than before. I was scared. I won't deny it. I sat bolt up in bed and presently there came from under the bed a huge black cat. He gave an unearthly 'meow!' which nearly froze my blood. I threw the Bowie knife at him with all my strength, but missed him, and it stuck in the floor with a thud. That scared the cat. He crouched in the corner ready for a spring, and growled, while his horrid green eye—he had but one—gleamed fire. The fight was on. I managed, however, to lay him out with the poker, and opening the shutter, threw him, limp and limber, into the street. Mice had doubtless made a nest in the straw and the cat was after the young mice; that's all. And, of course it was another cat—Pat had killed the first one. It was another remarkable coincidence, however, that this one, too, had a red scar on the face.

"At breakfast, Jenkins, an interne at the Charity Hospital across the street, rallied me on my haggard looks. 'I had an unpleasant experience last night,' said I, 'with a black cat that had gotten into my room and was annoying me;' and I related dramatically the incident.

"'Speaking of black cats,' said Jenkins; 'singular coincidence. A young woman was admitted to the hospital a short time ago, whose case was diagnosed hysterical convulsions. She said she was 'hanted by a cat,' and begged piteously to not let him come in; he had followed her, she said, for a year, everywhere. We supposed her mind was unsettled, and it was decided to send her to the asylum as soon as she got better. One day in the temporary absence of the nurse a black cat entered the ward, and jumping on the bed frightened her into convulsions in which she died.'

"'Doctor,' said Mrs. Butts, our landlady, who became very much excited, 'are you superstitious? Do you believe in 'hants?'

"'Why do you ask that?' said I.

"'Because your story and that of Mr. Jenkins remind me of a remarkable occurrence,' she said. 'I will relate it to you:

"'Last year there was a student by the name of Dick Darrell, boarded with me. He had the room you now occupy. He was the son of a rich sugar planter, and was the wildest, most reckless, dissipated fellow you ever saw. He squandered money, got in scrapes with the police; drove on the shell roads and smashed buggies; fired his pistol at dogs in the crowded streets, and was in all sorts of devilment. The students called him 'Dick Dare-devil.' He was a remarkably handsome fellow, too, tall, straight, as supple as a tiger and as active. His pretty curly head and clear, honest blue eye belied his real character. He was a devil.

"'I had a servant girl named Nora O'Riely. She was a typical Irish beauty, about twenty years of age; she had a willowy and voluptuous form, a pretty face and complexion, and her wealth of glossy bronze and gold hair would have adorned a queen. Her eyes were glorious, and her red ripe lips were full and tempting. She was, however, illiterate and superstitious, and a devout Catholic. Always neat and tidy in her calico and gingham dresses, she, with her merry, light hearted laugh, was like sunshine in the house. I was greatly attached to her. Dick fell desperately in love with her; perfectly infatuated. He could hardly keep his hands off of her. He tried every way to win her, but she hated him and was afraid of him. He offered her marriage, but she laughed at him. 'Do you think I am a fool?' she would say. Dick neglected his studies and would come to the house at any time of the day to see Nora. One day she was ironing in an outhouse, and Dick sought her there. I heard a scream and then a yell of pain and rage, and deep curses. Nora ran screaming to me, and clung to my skirts in fear. Dick came out cursing, with his handkerchief held to his face and went across to the hospital. In the scuffle, Nora, to defend herself, had thrust the hot iron to his face. It destroyed his left eye and resulted in an ugly red scar diagonally across the forehead and eye. After a month or more of confinement to the hospital under treatment, he went home and abandoned himself to drink, and speedily died of delirium tremens. He wrote Nora a letter saying: 'You have ruined my life and driven me to destruction. You might have saved me. I will curse you with my dying breath, and will haunt you, living and dead, through all time and eternity.' Shortly after we heard of Dick's death, a strange cat appeared on the place. It was a large black Tom cat, and it is a remarkable coincidence, that, like *your* cat, he was blind in the left eye and had a red scar on his face. He had an antipathy to Nora and growled or flew at her every time she went near him. She would cross herself and kiss the little crucifix she wore in her bosom suspended by a string from her neck. She declared it was Dick's 'hant' and she would cry as if her heart would break. Persecuted by the cat and distressed by this silly superstition, she ran away. The cat disappeared at the same time, and I have never seen either of them since. I did hear, through a rumor, that she was dead, but could never learn any particulars. The remarkable occurrence related by you, Mr. Jenkins, makes me suspect—or rather fear—that—perhaps— Did the girl at the hospital, Mr. Jenkins, have a red birth-mark on her left breast?' she asked, excitedly, and leaned breathlessly forward for his answer.

"Jenkins looked steadily at me for a moment and then said:

" 'No.'

" 'Thank God,' said Mrs. Butts, with a sigh of relief. 'Poor Nora, I wonder what became of her?'

"It was a pardonable lie," said the doctor, "it was Nora. I dissected her. Those were her bones under my bed. That is her skeleton."

As the doctor concluded his story the storm broke, and hail was rattling on the roof, making a fearful din. There was a blinding flash of lightning and an ear-splitting peal of thunder. The house trembled, the electric lights went out; there was a crash of glass, and with a wild, vibrant cry the "hant" leaped into the room.

"Damn that cat," said the doctor. "I'll have to kill him—*again*, I reckon."

I stood not on the order of my going. I went. Through the blinding hailstorm, I lit out for the hotel, a badly scared drug drummer.

When the drummer had finished the story I said: "You are the biggest liar in Texas. I don't believe a word of that spiel. You've been on a jag and dreamed it. Call again. I'm busy."

---

Inhalation of Medicated Vapor in diseases of the lungs, throat and bronchi has always been a favorite and successful method of treatment. Every physician of experience recognizes the value of it, and every person who has experienced the treatment appreciates its soothing and healing effects, especially in the harassing, exhausting and persistent cough that attends and follows la grippe, bronchitis, etc. By the judicious combination of remedies the physician is enabled to meet a wide range of therapeutic indications, and to treat with satisfaction by steam atomization not only the ordinary inflammations of the air passages, but consumption, asthma, etc. The desideratum has been to adapt an apparatus to meet the requirements, and many forms of "inhalers," and "atomizers" and "nebulizers" have been invented, offered to the physician, tried and generally abandoned—from the little pocket "inhaler" to the elaborate condensed air vaporizer. The several steam nebulizers, sold at from $1 to $5, have given best satisfaction, but they are too small for office practice and do not meet the requirements fully. Vaporization of medicated fluids under steam pressure is more effective than under air pressure.

It has been needed to equip the doctor's office with an apparatus by the use of which medicines in a state of fine subdivision can be deeply and safely inhaled into the lungs, even to the capillary tubes, and in a moist, warm vapor that will not provoke cough or distress the patient. Not only does the patient get the soothing effects of the application locally, but the systemic effects of the drug also, these surfaces being a short cut to the general circulation, absorption taking place rapidly. Creosote has always been a favorite drug in the treatment of consumption. It is supposed to be, and I presume is, germicidal; but it so deranges the digestion as to be a bar to its use. By the inhalation of creosoted steam vapor the benefits of the drug may be secured without detriment to the digestion, and it would seem that this method is especially adapted to the treatment of consumption. The dyspnœa of asthma may be spedily relieved by appropriate drugs, and as an auxiliary to a general hygiene and dietetic regimen, the process would seem to offer many advantages.

The demand for a suitable apparatus for the administration of drugs by the steam vapor process has been admirably met by the Terre Haute Inhalatorium Cabinet, manufactured by the Terre Haute Inhalatorium Cabinet Co. at Terre Haute, Ind. It is a zinc lined chamber, in which the patient is seated and the arrangement is such as to charge it with a certain per cent of medicated vapor which he breathes so many minutes, under the eye of the physician. There are several reservoirs into each, or any one of which, the medicine to be used is placed on a sponge and vaporized by a current of steam, generated by a suitable apparatus attached. The cabinet is used also for vapor baths, medicated with sulphur, iodine, mercury, tar or whatever is indicated, in certain forms of skin diseases, rheumatism, etc.

Dr. J. D. Roberts, the President of the company, exhibited the Cabinet at the Houston meeting of the State Medical Association. He has since placed a large number of the Cabinets in the offices of leading physicians, and he informs me that they are giving eminent satisfaction.

